# Serum ICAM-1 and VCAM-1 as markers of endothelial activation in ankylosing spondylitis and psoriatic arthritis: a cross-sectional study

**DOI:** 10.1186/s41927-026-00640-0

**Published:** 2026-04-02

**Authors:** Miroslav Markov, Maria Dimova, Simona Bogdanova, Trifon Chervenkov, Tsvetoslav Georgiev

**Affiliations:** 1 Department of Propaedeutics of Internal Medicine, Faculty of Medicine, Medical University of Varna, Varna, 9002 Bulgaria; 2 Clinic of Internal Medicine, University Hospital “St. Marina” – Varna, Varna, 9010 Bulgaria; 3 First Department of Internal Medicine, Faculty of Medicine, Medical University of Varna, Varna, 9002 Bulgaria; 4 Clinic of Rheumatology, University Hospital “St. Marina” – Varna, 1, Hristo Smirnenski blvd, Varna, 9010 Bulgaria; 5 Department of Medical Genetics, Faculty of Medicine, Medical University of Varna, Varna, 9002 Bulgaria; 6 Laboratory of Clinical Immunology, University Hospital “St. Marina” – Varna, Varna, 9010 Bulgaria

**Keywords:** Ankylosing spondylitis, Psoriatic arthritis, Endothelial dysfunction, VCAM-1, ICAM-1, Cardiovascular risk, Inflammation

## Abstract

**Background:**

Patients with ankylosing spondylitis (AS) and psoriatic arthritis (PsA) have increased cardiovascular risk beyond traditional factors. Endothelial activation is an early and potentially reversible stage of atherogenesis, and circulating adhesion molecules such as intercellular adhesion molecule-1 (ICAM-1) and vascular cell adhesion molecule-1 (VCAM-1) have been proposed as markers of vascular involvement in inflammatory diseases. This study aimed to compare serum ICAM-1 and VCAM-1 levels in patients with ankylosing spondylitis and psoriatic arthritis versus controls, and to examine their associations with disease activity, systemic inflammatory burden, cardiovascular risk profile, and treatment status.

**Methods:**

This cross-sectional study included 154 participants recruited between 2023 and 2024, including 83 patients with AS, 40 with PsA, and 31 controls. Disease activity was assessed using ASDAS-CRP (AS) and DAS28-CRP (PsA). Inflammatory markers, lipid profile, cardiovascular risk scores, and treatment history were recorded. Serum ICAM-1 and VCAM-1 concentrations were measured by enzyme-linked immunosorbent assay. Group comparisons used Kruskal–Wallis and Mann–Whitney U tests; correlations used Spearman or Pearson coefficients as appropriate. Multivariable linear regression was applied to identify independent predictors of VCAM-1.

**Results:**

Serum VCAM-1 and ICAM-1 levels did not differ significantly between AS, PsA, and controls. VCAM-1 showed positive correlations with C-reactive protein (ρ = 0.247, *p* = 0.002) and erythrocyte sedimentation rate (ρ = 0.163, *p* = 0.044), whereas ICAM-1 showed no significant associations with inflammatory, metabolic, or vascular parameters. In multivariable analysis, body mass index was the only independent predictor of VCAM-1 (β = 0.198, *p* = 0.027). In AS, higher disease activity defined by ASDAS-CRP > 2.1 was associated with higher CRP (*p* = 0.002), ESR (*p* = 0.04), and VCAM-1 concentrations (*p* = 0.002). Biologic-naïve patients had higher VCAM-1 levels than treated patients (*p* = 0.046), while those receiving Janus kinase inhibitors had lower VCAM-1 compared with biologic-naïve individuals (*p* = 0.014).

**Conclusions:**

Although ICAM-1 and VCAM-1 levels were comparable between patients and controls, VCAM-1 reflected inflammatory activity and treatment status, particularly in AS. VCAM-1 may represent a more sensitive marker of endothelial activation than ICAM-1 in treated spondyloarthritis.

## Introduction

Emerging evidence indicates that patients with ankylosing spondylitis (AS) and psoriatic arthritis (PsA) carry a substantially increased cardiovascular burden [1–3]. In AS, chronic inflammation may involve the conduction system and aortic root, resulting in conduction abnormalities and aortic regurgitation, while myocardial and diastolic dysfunction have also been documented [4–6]. Large cohort studies further show higher rates of ischemic heart disease, cerebrovascular events and cardiovascular mortality compared with the general population [1, 7–12].

Similar trends are observed in PsA, where increased overall and cardiovascular mortality, as well as elevated risks of myocardial infarction, stroke and heart failure, have been consistently reported [13, 14]. In both conditions, the cardiovascular burden is further amplified by a higher prevalence of traditional metabolic risk factors, including hypertension, diabetes, dyslipidaemia and metabolic syndrome [15–18]. Together, these data highlight the need for early cardiovascular risk assessment and targeted preventive strategies in patients with AS and PsA [19].

Atherosclerosis is currently recognized as a chronic inflammatory disease driven by a complex sequence of cellular and molecular events [20, 21]. One of the earliest steps involves increased endothelial permeability and loosening of endothelial junctions, allowing leukocyte entry into the subendothelial space. This process is tightly regulated by the activation of endothelial and leukocyte adhesion molecules (CAMs) in response to inflammatory stimuli [22–24].

Selectins mediate the initial rolling interaction between leukocytes and the endothelium, which is subsequently stabilized by intercellular adhesion molecule-1 (ICAM-1) and vascular cell adhesion molecule-1 (VCAM-1) in concert with leukocyte integrins [25, 26]. PECAM-1 (Platelet Endothelial Cell Adhesion Molecule-1) further facilitates transendothelial migration [27].

Following transendothelial passage, LDL particles become oxidized within the intima, promoting CAM expression and chemokine release, such as monocyte chemoattractant protein-1 (MCP-1), thereby enhancing monocyte recruitment and foam-cell formation-central processes in plaque development [28]. Activated foam cells subsequently release cytokines, chemokines and matrix-modifying enzymes, driving inflammation, extracellular matrix remodeling and vascular smooth-muscle proliferation. Persistent immune activation involving monocytes, T cells, B cells and mast cells contributes to plaque progression and destabilization through mediators such as TNF-α, IL-1β, IL-8 and MCP-1 [29, 30]. Beyond their pathogenic role, adhesion molecules also possess diagnostic and prognostic relevance. VCAM-1, ICAM-1 and L-selectin are upregulated in atherosclerotic lesions, and their soluble forms (sVCAM-1 and sICAM-1) are elevated in individuals with increased cardiovascular risk or established coronary disease [31, 32]. Soluble adhesion molecules are also dysregulated in a wide range of inflammatory, autoimmune and malignant conditions [33–35], and their elevation in inflammatory arthritis has been associated with increased atherosclerotic risk [36].

However, despite accumulating evidence linking adhesion molecules to endothelial dysfunction and cardiovascular risk, data directly comparing circulating sICAM-1 and sVCAM-1 levels between patients with ankylosing spondylitis, psoriatic arthritis and individuals without inflammatory joint disease remain limited and heterogeneous, particularly in contemporary cohorts reflecting real-world clinical practice. The present study aimed to evaluate serum levels of sICAM-1 and sVCAM-1 in patients with ankylosing spondylitis and psoriatic arthritis versus controls, and to explore their relationships with disease activity, systemic inflammatory burden, cardiovascular risk profile, and treatment status.

## Materials and methods

### Study population

This cross-sectional study included 154 participants recruited at University Hospital “St. Marina” – Varna between 2023 and 2024.

The study population comprised 83 patients with AS diagnosed according to the ASAS classification criteria, 40 patients with PsA fulfilling the CASPAR criteria, and 31 control healthy individuals.

All participants were aged ≥ 18 years and provided written informed consent prior to inclusion.

The number of controls was determined by strict eligibility criteria and availability of individuals without inflammatory joint disease or major comorbidities during the recruitment period.

Exclusion criteria included established ischemic heart disease, cerebrovascular or peripheral arterial disease, heart failure, arrhythmias, cardiomyopathies, diabetes mellitus or metabolic syndrome, chronic kidney disease stage ≥ 3, chronic obstructive pulmonary disease, active infection, malignancy, immunodeficiency, pregnancy, breastfeeding, or inability to undergo clinical evaluation.

Participants were recruited prospectively during routine clinical care in both outpatient and inpatient settings according to predefined inclusion and exclusion criteria. Control participants were screened to ensure the absence of significant comorbidities.

### Clinical and disease activity assessment

Clinical data were obtained through structured interview, physical examination, and review of medical records. Information collected included demographic parameters, smoking status, disease duration, current therapy, anthropometric measurements, blood pressure, and laboratory results. Disease activity was assessed using validated composite indices: ASDAS-CRP for patients with AS and DAS28-CRP for PsA, calculated from joint counts, patient-reported outcomes, and CRP. Both indices were interpreted according to established ASAS and EULAR thresholds.

### Laboratory measurements

Fasting venous blood samples were collected in the morning and processed in the accredited Clinical Laboratory of the hospital. The biochemical panel included standard inflammatory markers (C-reactive protein and erythrocyte sedimentation rate) and routine lipid profile parameters (total cholesterol, HDL-C, LDL-C, and triglycerides), all measured using standardized automated laboratory assays in the accredited clinical laboratory. The serum was separated, aliquoted, and stored at − 80 °C until further analyses.

### Adhesion molecule analysis

Serum concentrations of the endothelial dysfunction markers sICAM-1 and sVCAM-1 were determined in the Department of Clinical Immunology using commercial ELISA kits (Invitrogen BMS201 and BMS232, Thermo Fisher Scientific). Samples were processed following manufacturer protocols, including clotting, centrifugation, and appropriate dilution prior to analysis. Optical density was measured at 450 nm with reference correction, and concentrations were calculated based on four-parameter logistic regression standard curves, applying the corresponding dilution factors.

### Derived indices

Several derived cardiovascular variables were calculated for all participants. Body mass index (BMI) was computed from measured height and weight. Cardiovascular risk estimation was performed using the Framingham Risk Score, SCORE2 (for individuals < 70 years), and SCORE2-OP (for individuals ≥ 70 years), interpreted according to ESC-calibrated thresholds for Bulgaria.

### Statistical analysis

Statistical analysis was performed using IBM SPSS Statistics version 27.0 [1]. Normality of distribution was assessed with the Shapiro–Wilk and Kolmogorov–Smirnov tests. Continuous variables were expressed as mean ± standard deviation for normally distributed data or median with interquartile range for non-normally distributed data, while categorical variables were reported as counts and percentages. Group comparisons were performed using the Kruskal–Wallis test for three-group analyses and the Mann–Whitney U test for pairwise comparisons. Categorical data were compared using Pearson’s chi-square test. Correlations between continuous variables were evaluated using Pearson’s and Spearman’s correlation coefficients as appropriate. A two-tailed p-value < 0.05 was considered statistically significant. Given the observed baseline differences between groups, particularly in age and sex distribution, subgroup comparisons were interpreted cautiously and considered exploratory. Multivariable linear regression analysis was performed to identify independent predictors of VCAM-1, including clinically relevant covariates, while acknowledging that residual confounding may persist due to the cross-sectional design.

### Ethical approval

The study was conducted according to the principles of the Declaration of Helsinki. Ethical approval was obtained from the Ethics Committee of the Medical University – Varna (Protocol №133/22.06.2023), and all participants signed informed consent prior to inclusion.

## Results

### Baseline characteristics and cardiometabolic profile

In the baseline demographic assessment, patients with psoriatic arthritis (PsA) were the oldest group, followed by the controls and those with ankylosing spondylitis (AS). A marked sex imbalance was observed across groups (*p* = 0.002), with a predominance of men among AS patients (66.3%) and women among PsA participants (67.5%), while the control group showed a more even distribution. Body mass index was comparable across all three cohorts, and no significant differences were found in smoking prevalence (*p* = 0.497). Arterial hypertension was most frequent in the PsA group (47.5%), although differences versus AS (27.7%) and controls (35.5%) did not reach statistical significance (*p* = 0.095) (Table 1).

**Table 1:** Demographic characteristics, lipid profile, cardiovascular risk scores, and adhesion molecules

Parameter	AS (*n* = 83)	PsA (*n* = 40)	Controls (*n* = 31)	*p*-value
Demographic Characteristics				
Mean age (years)	47.05 ± 10.62	54.03 ± 11.01	48.35 ± 11.27	**0.004**
Sex (% male)	66.3%	32.5%	54.8%	**0.002**
Arterial hypertension (%)	27.7%	47.5%	35.5%	0.095
Smoking (% smokers)	55.4%	65.0%	64.5%	0.497
BMI (kg/m²)	27.75 ± 5.31	28.47 ± 5.64	26.73 ± 4.78	0.630
Lipid Profile				
Total cholesterol (mmol/L)	5.38 ± 1.21	5.44 ± 1.17	5.16 ± 0.86	0.705
Triglycerides (mmol/L)	1.48 ± 1.46	1.43 ± 0.78	1.58 ± 0.97	0.382
LDL (mmol/L)	3.25 ± 1.08	3.29 ± 0.94	2.93 ± 0.80	0.266
HDL (mmol/L)	1.44 ± 0.41	1.49 ± 0.37	1.54 ± 0.44	0.497
non-HDL (mmol/L)	3.95 ± 1.12	3.93 ± 1.11	3.62 ± 0.86	0.397
Cardiovascular Risk Scores				
Framingham Risk Score (%)	5.43 ± 4.91	5.27 ± 4.34	6.76 ± 4.48	0.648
SCORE2 (%)	8.61 ± 6.72	11.52 ± 7.69	8.89 ± 6.24	0.146
Adhesion Molecules				
ICAM-1 (ng/mL)	460.93 ± 145.52	463.74 ± 127.97	492.91 ± 130.15	0.386
VCAM-1 (ng/mL)	813.94 ± 262.98	927.15 ± 377.20	852.10 ± 214.57	0.097

The lipid profile showed broadly similar values between AS, PsA, and control participants. Total cholesterol ranged between 5.16 and 5.44 mmol/L, LDL between 2.93 and 3.29 mmol/L, HDL between 1.44 and 1.54 mmol/L, and triglycerides between 1.43 and 1.58 mmol/L (all *p* > 0.05).

**Fig. 1:**
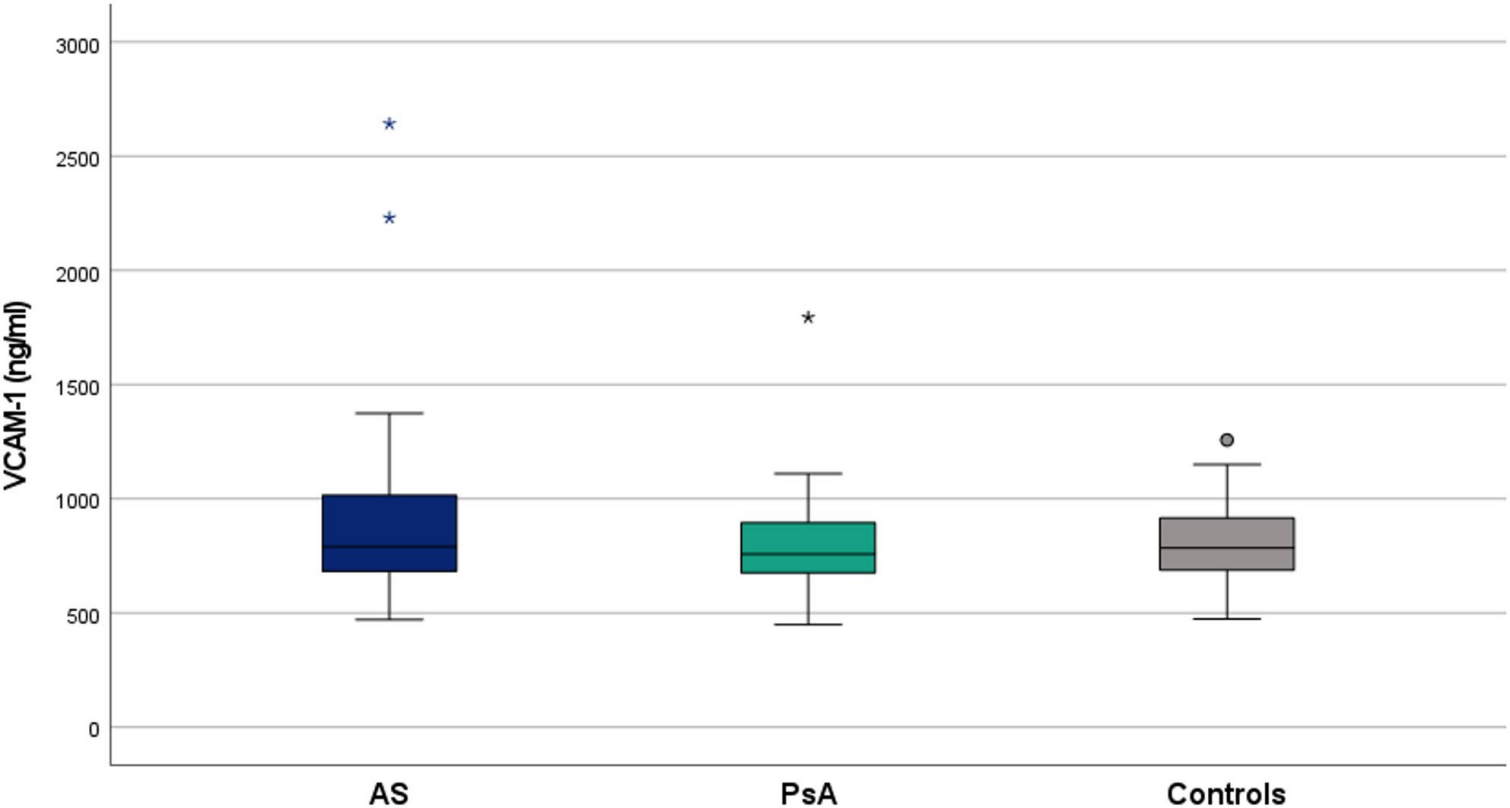
Serum VCAM-1 levels across study groups. Abbreviations: AS: ankylosing spondylitis, PsA: psoriatic arthritis, VCAM-1: vascular cell adhesion molecule-1

**Fig. 2:**
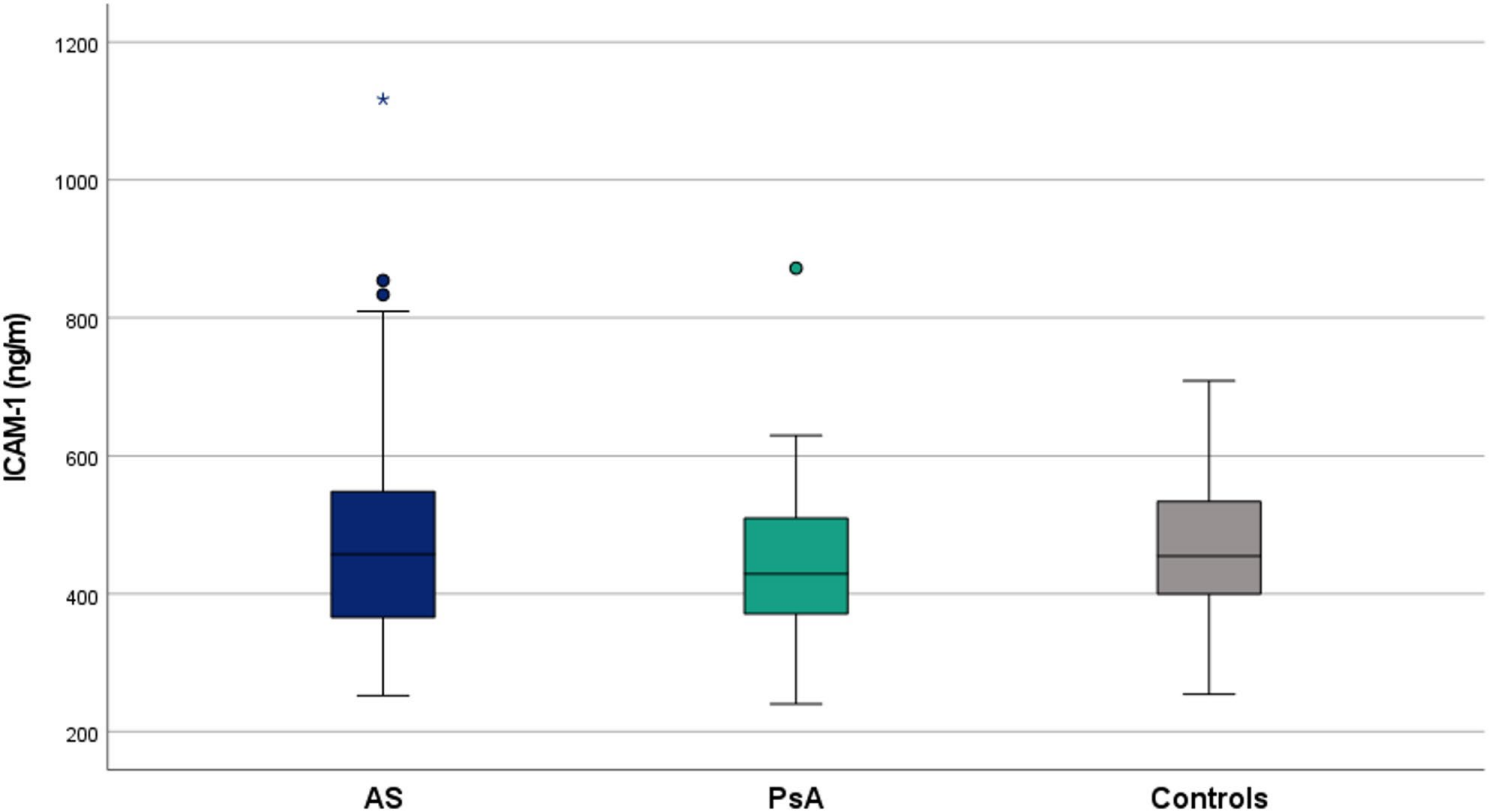
Serum ICAM-1 levels across study groups. Abbreviations: AS: ankylosing spondylitis; PsA: psoriatic arthritis; ICAM-1: intercellular adhesion molecule-1

In contrast to lipid parameters, cardiovascular risk scores displayed more variation. SCORE2 was numerically highest in PsA patients, compared with AS and controls, whereas the Framingham risk score remained comparable among the three cohorts. Regarding endothelial activation, mean VCAM-1 concentrations were highest in PsA (927.15 ± 377.20 ng/mL) and moderately elevated in AS (813.94 ± 262.98 ng/mL) and controls (852.10 ± 214.57 ng/mL). Mean ICAM-1 levels were similar across groups (AS 460.93 ± 145.52; PsA 463.74 ± 127.97; controls 492.91 ± 130.15). However, no statistically significant differences were observed between AS, PsA, and control groups for either VCAM-1 (*p* = 0.097) or ICAM-1 (*p* = 0.386) levels (Figs. 1 and 2).

### Inflammatory activity, disease severity, and endothelial activation

Inflammatory activity differed markedly between groups. AS patients demonstrated the highest median ESR (22 mm/h) and CRP (6.3 mg/L), followed by PsA patients (ESR 16 mm/h; CRP 4.1 mg/L), while controls exhibited values within the normal range (ESR 9 mm/h; CRP 1.8 mg/L). Disease activity indices reflected predominantly low to moderate activity: ASDAS-CRP averaged 2.30 ± 1.30 and BASDAI 2.83 ± 1.96 in AS, whereas DAS28 in PsA was 2.82 ± 0.83.

VCAM-1 demonstrated a broader pattern of associations compared with ICAM-1, showing correlations with markers of systemic inflammation. Although VCAM-1 showed significant positive correlations with CRP (ρ = 0.247, *p* = 0.002) and ESR (ρ = 0.163, *p* = 0.044) in univariate analysis, these associations did not persist after multivariable adjustment. In the fully adjusted linear regression model, BMI emerged as the only independent predictor of VCAM-1 (β = 0.198, *p* = 0.027; 95% CI for B: 1.34–21.41), while inflammatory markers lost statistical significance. This suggests that the relationship between systemic inflammation and endothelial activation may be partly mediated by metabolic factors rather than representing a direct independent effect.

ICAM-1 did not show statistically significant correlations with inflammatory markers (ESR: rho = -0.032, *p* = 0.697; CRP: rho = 0.093, *p* = 0.254), lipid profile, or vascular indices. Taken together, these findings suggest that VCAM-1, rather than ICAM-1, reflects a composite signal of systemic inflammation and cardiovascular risk in patients with inflammatory arthropathies.

### Impact of disease activity and age stratification on vascular and inflammatory parameters

When stratifying participants into age categories (≤ 45, 46–60, > 60 years), no statistically significant differences were identified for inflammatory markers, lipid parameters or adhesion molecules (all *p* > 0.05), indicating that age alone did not substantially influence these characteristics within the studied population.

Participants with inflammatory arthropathies were stratified according to established disease activity thresholds: DAS28 ≤ 3.2 versus > 3.2 for psoriatic arthritis (PsA), and ASDAS ≤ 2.1 versus > 2.1 and BASDAI < 4 versus ≥ 4 for ankylosing spondylitis (AS).

In contrast, disease activity in AS demonstrated clearer associations. When stratified by ASDAS, patients with high activity exhibited significantly higher CRP (*p* = 0.002), ESR (*p* = 0.040) and VCAM-1 concentrations (*p* = 0.002). These findings highlight the relationship between elevated systemic inflammation, endothelial activation. No significant differences were observed in lipid profile, Framingham, or anthropometric and hemodynamic variables.

Activity stratification using BASDAI did not yield statistically significant differences for any clinical, biochemical, or vascular parameters. This divergence between ASDAS and BASDAI is consistent with the more subjective nature of BASDAI and its weaker alignment with objective inflammatory or vascular markers.

### Therapeutic subgroup comparisons and associations with endothelial markers

Comparisons between therapeutic subgroups were conducted as exploratory analyses to further characterise treatment-related differences. Overall comparisons across anti-TNF, anti-IL-17, JAK inhibitor and biologic-naïve subgroups did not demonstrate substantial variation in the principal clinical, inflammatory, metabolic or endothelial parameters. Pairwise comparisons between the three targeted therapy groups (anti-TNF, anti-IL-17 and JAK inhibitors) were performed using the Mann–Whitney U test.

Clinical, inflammatory, metabolic and endothelial parameters across biologic-naïve patients and those receiving anti-TNF, anti-IL-17 or JAK inhibitor therapy are summarised in Table 2.

**Table 2:** Comparison of clinical, inflammatory, metabolic and endothelial markers across treatment subgroups

Variable	Biologic-naïve (*n* = 20)	Anti-TNF (*n* = 58)	Anti-IL-17 (*n* = 32)	JAK inhibitor (*n* = 13)
Age (years)	45.55 ± 11.17	48.67 ± 11.29	50.72 ± 10.37	54.54 ± 11.53
Disease duration (years)	8.34 ± 8.06	10.12 ± 6.46	9.48 ± 6.77	8.31 ± 4.09
ESR (mm/h)	39.20 ± 28.23	37.19 ± 23.26	33.21 ± 22.53	24.14 ± 12.64
CRP (mg/L)	9.84 ± 16.11	7.60 ± 12.55	6.63 ± 18.06	5.21 ± 5.43
Total cholesterol (mmol/L)	5.44 ± 1.10	5.33 ± 1.17	5.44 ± 1.40	5.56 ± 0.94
Triglycerides (mmol/L)	1.38 ± 0.95	1.56 ± 1.43	1.27 ± 0.92	1.67 ± 1.76
LDL-C (mmol/L)	3.37 ± 0.87	3.20 ± 1.06	3.30 ± 1.13	3.30 ± 0.97
HDL-C (mmol/L)	1.47 ± 0.37	1.40 ± 0.36	1.53 ± 0.51	1.49 ± 0.32
Framingham risk score (%)	3.68 ± 4.00	6.18 ± 4.94	5.27 ± 4.79	4.67 ± 4.11
SCORE2 (%)	6.25 ± 4.70	9.75 ± 7.74	11.27 ± 7.42	9.61 ± 5.65
BMI (kg/m²)	26.53 ± 3.81	28.35 ± 5.61	28.20 ± 6.01	28.06 ± 5.23
sICAM-1 (ng/mL)	455.14 ± 155.90	467.21 ± 123.13	496.50 ± 181.16	434.38 ± 113.66
sVCAM-1 (ng/mL)	955.30 ± 261.76	854.75 ± 362.16	844.11 ± 275.33	747.20 ± 151.54

In a focused two-group comparison, patients receiving anti-TNF therapy demonstrated higher ESR (*p* = 0.050) and pulse pressure (*p* = 0.015) than those treated with JAK inhibitors, whereas arterial stiffness measures remained comparable.

When comparing anti-TNF–treated with biologic-naïve patients, the anti-TNF group exhibited longer disease duration (*p* < 0.001) and higher pulse pressure (*p* = 0.041). Conversely, biologic-naïve individuals displayed higher VCAM levels (*p* = 0.046) and higher SCORE2 values (*p* = 0.020), however, these findings should be interpreted cautiously, as subgroup comparisons were exploratory and not adjusted for baseline cardiovascular risk factors. JAK inhibitor–treated patients had significantly lower VCAM concentrations compared with biologic-naïve patients (*p* = 0.014), which may suggest a potential association, although causal or treatment-related effects cannot be inferred from this cross-sectional analysis.

Comparison between anti-TNF and IL-17 inhibitors identified higher CRP (*p* = 0.015), ASDAS-CRP (*p* = 0.032), triglycerides (*p* = 0.049) in the anti-TNF subgroup, while vascular indices, adhesion molecules, and cardiovascular risk scores did not differ significantly. Differences between IL-17 inhibitors and JAK inhibitors were confined to treatment duration (*p* = 0.005), with no significant variation in inflammatory, metabolic parameters.

## Discussion

The present study evaluated endothelial activation in patients with AS and PsA by analysing circulating adhesion molecules ICAM-1 and VCAM-1-together with inflammatory, metabolic and vascular parameters. The main findings were that serum ICAM-1 and VCAM-1 levels were comparable between patients and controls, while VCAM-1 showed associations with inflammatory burden, disease activity, and treatment status, particularly in ankylosing spondylitis. These results suggest that VCAM-1 may reflect dynamic inflammatory and metabolic influences on endothelial activation even in the absence of overt between-group differences. Clinically, this highlights the potential role of adhesion molecules as complementary biomarkers of vascular risk assessment in real-world spondyloarthritis populations.

Across diagnostic groups, mean VCAM-1 levels were numerically highest in PsA, followed by controls and AS, whereas ICAM-1 values were largely comparable. These findings differ from earlier reports showing consistently elevated adhesion molecule levels in untreated or more active SpA populations. Prior studies demonstrated higher ICAM-1 in patients with spondyloarthritis and its correlation with inflammatory markers [37], elevated ICAM-1 and VCAM-1 in AS associated with disease activity and impaired endothelial function measured by flow-mediated dilation [38], and increased adhesion molecules in early PsA with positive associations to CIMT [39]. Additional investigations in PsA also reported raised ICAM-1 and VCAM-1 linked to endothelial dysfunction and subclinical atherosclerosis [40–42].

The lack of intergroup differences in our cohort may be explained by the heterogeneous inflammatory burden observed across diagnostic groups. Disease activity ranged from low activity in psoriatic arthritis to predominantly moderate-to-high activity in ankylosing spondylitis, which may have reduced clear contrasts in endothelial activation markers between groups. In addition, a large proportion of patients were receiving biologic or targeted synthetic therapies, which could attenuate adhesion molecule expression and further diminish intergroup variability. Soluble adhesion molecules are also influenced by metabolic and anthropometric factors, as supported by the independent association between VCAM-1 and BMI in the multivariable model. Finally, the cross-sectional design and modest subgroup sizes may have limited the statistical power to detect subtle differences.

Despite the absence of between-group differences, within-disease analyses revealed clinically relevant associations. In AS, VCAM-1 was higher in patients with greater disease activity, consistent with mechanistic data showing that inflammatory cytokines upregulate endothelial VCAM-1 expression. Positive correlations between VCAM-1 and CRP/ESR in our study parallel findings from previous reports [38, 43], reinforcing the link between systemic inflammation and endothelial activation.

In contrast, ICAM-1 showed no consistent associations with clinical, metabolic or vascular parameters. Although ICAM-1 has been associated with hypertension, dyslipidaemia and smoking in AS [44], and with inflammatory markers in PsA [39, 41, 42], such relationships were not observed in our cohort-likely due to milder inflammation and treatment-modulated disease.

Therapeutic subgroup analyses provided additional insights. Patients receiving biologic or targeted synthetic therapies-especially TNF inhibitors and JAK inhibitors-demonstrated lower VCAM-1 levels compared with biologic-naïve individuals. This is consistent with evidence showing improvement in endothelial function and reductions in adhesion molecules after TNF blockade [45–47]. Although clinical data on JAK inhibitors in SpA are limited, experimental studies have shown suppression of endothelial VCAM-1 expression following JAK-STAT pathway inhibition.

Overall, the findings suggest that VCAM-1 showed stronger associations with inflammatory and clinical parameters compared with ICAM-1 within this cohort. These findings should be interpreted with caution given baseline demographic imbalances between groups, which may have influenced some subgroup comparisons despite multivariable modelling. Although major between-group differences were not detected, the internal gradients within AS and the therapy-related reduction in VCAM-1 levels support a model in which endothelial dysfunction is dynamic and modifiable. Longitudinal studies are needed to determine whether modulation of VCAM-1 translates into improved vascular outcomes and reduced long-term cardiovascular risk.

### Limitations of the study

This study has several limitations that should be acknowledged. Its cross-sectional design does not allow causal inference, and the generally low inflammatory activity-together with the high proportion of patients receiving biologic or targeted therapies-may have attenuated differences in adhesion molecule levels. Some therapeutic subgroups, particularly JAK inhibitor–treated and biologic-naïve patients, were relatively small, increasing the risk of type II error. The control group was not strictly matched for age and sex, which represents an important methodological limitation, particularly in the context of biomarker studies assessing cardiovascular risk. Differences in major demographic characteristics may have influenced circulating adhesion molecule levels and should be considered when interpreting the results. Future studies with larger and better-matched control populations are warranted to confirm these findings. Finally, endothelial function was assessed only through circulating adhesion molecules without complementary functional testing, and biomarker measurements were performed at a single time point.

In conclusion, our findings indicate that while overall ICAM-1 and VCAM-1 levels do not differ substantially between AS, PsA and controls, VCAM-1 remains a sensitive marker of endothelial activation linked to inflammatory burden and treatment status, underscoring its potential relevance in assessing early vascular involvement in inflammatory arthropathies.

## Data Availability

The datasets generated and/or analysed during the current study are not publicly available due to the inclusion of sensitive personal and clinical data but are available from the corresponding author on reasonable request.

## Abbreviations

AS: Ankylosing spondylitis
ASAS: Assessment of SpondyloArthritis International Society
ASDAS: Ankylosing Spondylitis Disease Activity Score
BASDAI: Bath Ankylosing Spondylitis Disease Activity Index
BMI: Body mass index
CAMs: Cell adhesion molecules
CASPAR: Classification Criteria for Psoriatic Arthritis
COPD: Chronic obstructive pulmonary disease
CRP: C-reactive protein
DAS28: Disease Activity Score in 28 joints
ELISA: Enzyme-linked immunosorbent assay
ESR: Erythrocyte sedimentation rate
HDL-C: High-density lipoprotein cholesterol
ICAM-1: Intercellular adhesion molecule-1
IL: Interleukin
JAK: Janus kinase
LDL-C: Low-density lipoprotein cholesterol
MCP-1: Monocyte chemoattractant protein-1
PsA: Psoriatic arthritis
SCORE: Systematic Coronary Risk Estimation
sICAM-1: Soluble intercellular adhesion molecule-1
sVCAM-1: Soluble vascular cell adhesion molecule-1
TNF: Tumor necrosis factor
VCAM-1: Vascular cell adhesion molecule-1
